# A Potential ‘Curative’ Modality for Crohn’s Disease---Modeled after Prophylaxis of Bovine Johne’s Disease

**Published:** 2012-05-31

**Authors:** Robert E Click

**Affiliations:** N8693 1250 Street, River Falls, WI. 54022, USA

**Keywords:** Bovine Johne’s disease, *Dietzia* probiotic, Mycobacterium avium, MAP, Diabetes, Multiple sclerosis, IBS, Ulcerative colitis, Crohn’s disease, Diarrhea, Therapy

## Abstract

A naturally occurring, gastrointestinal disorder of ruminants (Johne’s disease) is a chronic, debilitating, lethal disease. The causative agent is *Mycobacterium avium* subspecies *paratuberculosis* (MAP). Exposure that leads to disease occurs primarily in utero and/or during the neonatal period. Outside a dietzia probiotic treatment, there are no preventive/curative therapies. Interestingly, MAP is at the center of a controversy as to its role (cause of, perpetuate of, innocent bystander) in Crohn’s disease, ulcerative colitis, irritable bowel syndrome, diabetes, sarcoidosis, Blau syndrome, and multiple sclerosis—diseases in which the incidence of systemic MAP is higher than that in the general population. Conventional therapeutic modalities, including biologic agents, for the majority of these diseases are, in general, directed at curtailing processes that are an intricate part of inflammation, with goals to induce and maintain remission. Most possess side effects of varying severity, lose therapeutic value, and more importantly, few are directed at prevention, attainment of long lasting remissions or cures, and essential none at reduction/elimination of MAP.

This report presents a rationale for how/why *Dietzia* subsp. C79793-74 should be clinically evaluated for efficacy in patients with IBD. Arguments are based on previous studies that demonstrated (a) clinical similarities of Johne’s disease and Crohn’s disease, (b) inhibition of growth of MAP by Dietzia under specific culture conditions, (c) safe usage for extended daily treatments of adult cattle (up to 24 months), and (d) when used as a probiotic, curtailed diarrhea and cured 40% of adult cattle with early stage *paratuberculosis.*

## Introduction

Crohn’s disease (CD), ulcerative colitis (UC), and some cases of irritable bowel syndrome (IBS), sub-phenotypes of inflammatory bowel disease (IBD), are generally considered a consequence of gastrointestinal inflammation of unknown etiology. Based primarily on histopathology, genetic predisposition, and effective (and ineffective) therapeutic treatments, the clinical manifestations have been postulated to be a consequence of an unregulated immune response due to: lack of regulator cell function, deletion of antigen-reactive cells (oral mucosal tolerance), loss of epithelial barrier architecture, and most recently, immune deficiency/dysfunction [1–3]. Conventional therapy includes anti-inflammatory and immunosuppressive drugs which are directed primarily at achieving symptomatic relief and preventing relapses: most are aimed at suppressing inflammatory cascade processes that are likely end-stage events. The agents include steroids, nonsteroidal anti-inflammatory drugs, and more recently, biologic agents [4–6]. All have drawbacks [7,8], in that many fail over prolonged periods, serious adverse events occur, use may be discontinued, and some are associated with an increased risk of lymphoma, cancer, and even death [9,10]. Even though the current regimens have improved control of symptoms associated with IBD, since they are not directed at treating the (undefined) primary cause, it cannot be over emphasized that prevention, permanent remission, and more importantly, cures still remain unmet needs. Moreover, current remedies are having little impact on the rising incidence and prevalence [2].

Recently it was reported that *Dietzia* subsp. C79793-74, when used as a probiotic had prophylactic and therapeutic benefits for Johne’s disease [11–15], a symptomatic IBD of cattle [16–19]. The primary goal of the present report is to outline how/why prophylactic benefits obtained with this bacterium could similarly curtail diarrhea of and/or cure human IBDs, with an emphasis on Crohn’s disease. To this end, extensive studies previously reported will not be reiterated, but examples will be used to support similarities and potential benefits.

## Materials and Methods

### Experimental design

Paratuberculosis-free and-positive animals (see below for definition of each), under the author’s ownership and management, were housed together in a tie-stall facility (each animal tied in their own space) as a single dairy herd. Since previous goals were to have a treatment modality amenable for use on farms (and not in a research laboratory), all aspects of the research were conducted using standard operating conditions employed by normal dairy farms. At any given time, the herd was comprised of 50–60 adult dairy cows. Once any *paratuberculosis* parameter of an animal was defined as positive, treatment with Dietzia was, or was not, initiated. Thereafter, various parameters were monitored over her remaining lifetime. It was predetermined, based on cost considerations and as a means to define effective doses for different stages of disease, that the dose of Dietzia would be adjusted for each individual based only on changes of early clinical symptoms (fecal consistency and appetite) and not on changes in any *paratuberculosis*- specific test parameter. Local veterinarians humanely euthanized recumbent, emaciated and/or cachectic end-stage paratuberculosis animals when they no longer could get up and stand on their own by intravenous injection of a sodium pentobarbital solution (Fatal Plus, 6 gm/ml, Vortech Pharmaceuticals, Dearborn, MI) at 1 ml/4.5 kg body weight. Animals that showed potential life-threatening non-Johne’s disease ramifications were also humanely terminated.

### Animals

Animals in the study were adult dairy cows in their first through third lactation. Severity of disease was defined by the following classification [20]: animals that were asymptomatic, and test-parameter negative were classified Stage I (considered paratuberculosis, disease-free); those asymptomatic, and either ELISA and/or fecal positive were classified as being at Stage II. Stage III animals had mild clinical symptoms (diarrhea, off feed) and were either ELISA and/or fecal positive, whereas, Stage IV animals were emaciated and end-stage, irrespective of whether they were ELISA and/or fecal negative/positive (essentially all were off feed, had pipestream diarrhea, and most were fecal shedding MAP). All diseased animals were purchased from seven local, well-managed, moderately high-prevalence herds over a five year period. These animals were identified at dry-off (2 months prior to their predicted calving date, milking was discontinued) by paratuberculosis-specific ELISAs (test-laboratories were chosen by the owners and their veterinarians, most were affiliated with the state of WI). Conformation that an ELISA-positive animal was Johne’s disease positive was based on whether she (a) eventually tested positive for a different parameter (fecal shedding or serum AGID), (b) developed end-stage (Stage IV) clinical disease, (c) was determined to have paratuberculosis via complete autopsy, and/or (d) as a last resort, tested ELISA positive multiple times. As reported previously using these criteria [12,13], an ELISA value considered positive (OD >1.4) by Allied Monitor, Inc. (Fayette, MO), was a very accurate indicator of paratuberculosis. Allied Monitor is a USDA- and NVSL-approved laboratory that specializes in assays for Johne’s disease.

### Dietzia

Dietzia (partially characterized and originally misclassified as *Mycobacterium gardonae*) was isolated from fecal material of a cow that tested both sero- and fecal-positive for paratuberculosis [21]. It was reclassified as *Dietzia* based on its 16S rRNA sequence (performed by MIDI Labs, Inc., Newark, DE); considered the gold standard for bacterial identification [22]. It was grown for 4–5 days at 29°C in 75-liter fermenters at the University of Minnesota Biotechnology Institute (St. Paul, MN) in fructose supplemented tryptic soy broth. Batches were centrifuged, washed and concentrated 20-fold prior to storage as 45 ml aliquots at −80°C (long term) or −20°C (short term). New lots were prepared as needed, approximately every 2–3 months. The number of colony forming units/ml was determined prior to use. Once thawed, aliquots were maintained at 4°C for up to 7 days only. *Dietzia* treatment was always initiated after an animal was detected ELISA positive. As the goal was to assess the effect of intervention rather than confirm findings of others that untreated paratuberculosis animals eventually succumb with disease, more paratuberculosis-positive animals were treated (n = 49) than not treated (n = 9). Based on preliminary dosage experiments, small cows (Jerseys and Jersey x Holstein crosses), and large cows (Holsteins and Guernseys) were initially treated by supplementing their morning feed with Dietzia at a minimally effective daily dose of 2–3×10^11^ and 4–5×10^11^ cfu, respectively. The dose was increased if an animal showed clinical signs of disease and lowered if remission was achieved.

### Body and milk weights, serum and fecal protocols

Changes in body weight and milk produced (the latter officially measured by DHIA technicians) were initially used to monitor progression/regression of disease. They were eventually discontinued because changes in each paralleled one another and because both fecal composition and changes in appetite predicted onset of clinical disease much earlier. Test parameters were determined on fecal and blood samples. Fecal material was collected directly from the rectum using individual disposable gloves and blood was obtained aseptically from the tail vein. After transferring to coded, sterile containers, they were sent chilled on the day of collection to Allied Monitor. The majority, but not all, fecal and serum samples were obtained concurrently. All serum ELISA and AGID assays and fecal MAP cultures were performed upon receipt. Assessment of the validity, sensitivity and specificity of all these assays was reported previously [12].

The ELISA specific for MAP was performed using a crude, soluble, MAP protoplasmic antigen prepared by Allied Monitor. Test sera were preabsorbed with *Mycobacterium phlei.* Split-sample repeatability, as well as duplicate samples, varied less than 5 percent of the mean. The content of each well was read at a single wavelength (405 nm). ELISA values were calculated by dividing the test-sample OD by a value equivalent to ¼ the OD of a standard reference positive serum (range 0.13–0.14), and interpreted as suggested by Allied: </=1.4 OD = Negative and all >1.4 ODs = positive.

Fecal samples of 2 g were weighed into 50 ml sterile conical centrifugation tubes containing 35 ml of sterile 0.75% hexadecylpyridinium chloride (HPC) (Sigma Aldrich, St. Louis, MO). Tubes were shaken for 30 min by a horizontal shaker, after which the contents allowed to settle in upright tubes for 2 h. Approximately 2 ml of supernatant, just above the sedimentation layer, was removed using a sterile transfer pipette. The loaded pipette containing supernatant was placed upright into a sterile culture tube (20 × 75 mm) at room temperature for overnight decontamination. Triplicate Herrold’s egg yolk (HEY) agar slants containing 50 mg each Naladixic acid, Vancomycin and Amphotericin B (Becton Dickinson and Company, Sparks, MD) with mycobactin-J and one without (to evaluate mycobactin dependency of any colonies) were inoculated with four drops (0.1 ml) of the decontaminated supernatant. Slants were maintained at 37°C in a horizontal position for 1 week, after which they were placed in the upright position and incubated for a total of 13 weeks. Evaluation/enumeration of slightly raised white-yellow colonies by Allied Monitor for typical acid fastness and morphological appearance of MAP of the triplicates was recorded and summed together. Slants with so many colonies that made it impossible to accurately count were estimated and expressed as >100 cfu and are shown in the Tables and Figures as >300 cfu (>100 × 3 slants).

### Postmortem analysis

As an additional means to document Johne’s disease, pathological postmortem analysis and culture-determination of MAP in tissues was done on randomly chosen cows at the University of Minnesota Veterinary School Diagnostic Laboratory (St. Paul, MN). Summation of the University’s standard basic necropsy, tissue histopathology of multiple organs, culture/PCR, bacteriology, parasitology, serology and molecular diagnostics was used to confirm positive/negative status only; none of these assays were intended to define specific aspects, category of disease, or be compared to ante-mortem parameters.

### Statistical methods

Survival time was defined as the number of months from the date at which an ELISA value was first detected positive (or if always negative, the first test-date) until their demise. Linear longitudinal best-fit analysis used to estimate trends in ELISA values for each animal was previously found to be the best indicator of progression/regression of disease [12,13]. The Mann-Whitney Test was used to assess differences in mean values. For all comparisons, P-values less than 0.05 were considered statistically significant.

## Results

**Figure 1:**
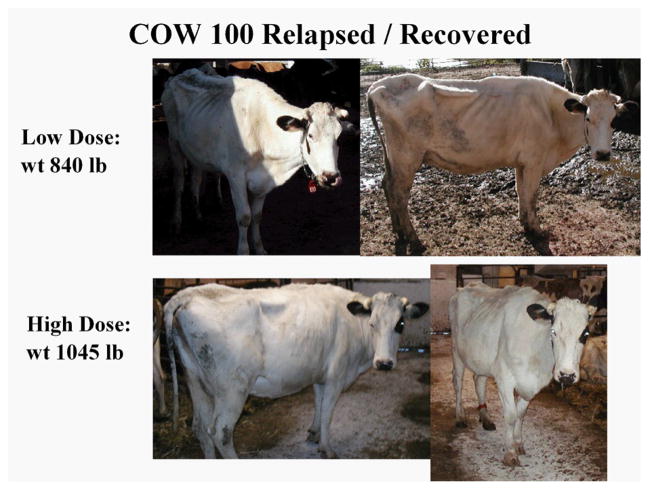
Photos of cow #R100 with late-stage clinical disease and after Dietzia- induced recovery.

**Figure 2:**
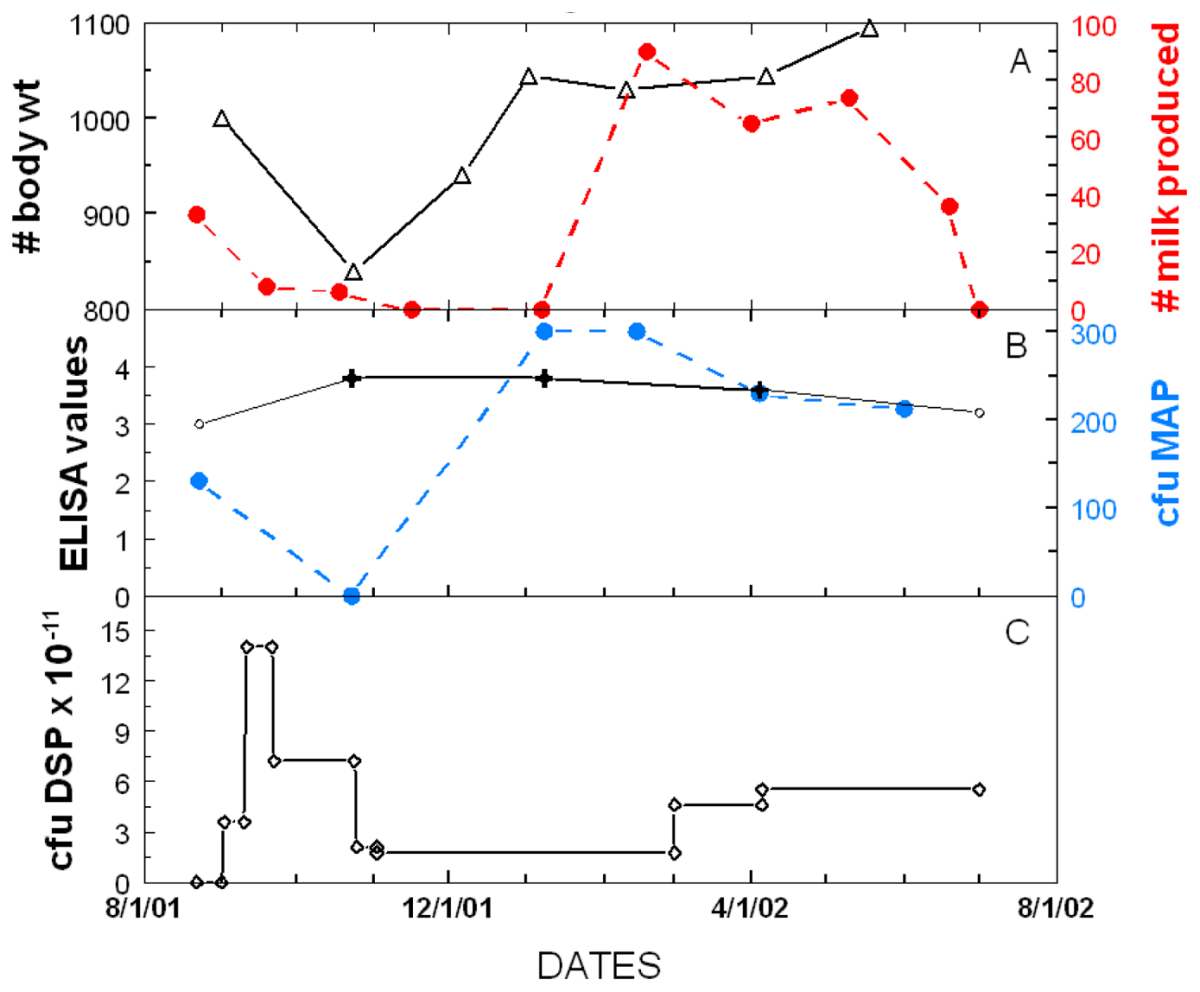
Paratuberculosis parameters of cow #R100 at different doses of Dietzia.

The upper panels of Figure 1 show a representative example of a paratuberculosis cow (#R100) with clinical Stage IV disease [20] that developed 55 days after initiation of a low-dose Dietzia treatment. The dose was then increased 4-fold; the bottom panels show her 70 days later at Stage II, asymptomatic. Other changes (body weight and milk production) that are associated with these visual changes are shown in Figure 2A. As shown, while on the initial low dose, body mass and milk production both declined. Following Dietzia adjustment (milking was discontinued at this time because she was due to calve in 2 months), body mass increased. In addition, fecal shedding of MAP declined; whereas, the ELISA OD values remained unchanged (Figure 2B) and she seroconverted to AGID positive (a test that indicates a more advanced stage of disease than ELISA values). Upon remission, the Dietzia dose was lowered (Figure 2C), after which fecal shedding increased dramatically (fecal data was reported previously in reference 8). At this time her weight remained stable, and ELISA/AGID serological profiles remained unchanged. She calved at 160 days of treatment and 60 days later began to show clinical signs of relapsing. At this time, the dose of Dietzia was modestly increased, after which both shedding and serological parameters declined slightly. Unfortunately, the new dose was ineffective and she died 180 days after calving with end stage IV, clinical disease. At autopsy, she was confirmed to harbor MAP in multiple tissues and showed pathological changes characteristic of paratuberculosis. The extent of fecal shedding was extremely variable over the course of treatment, changes appearing to be associated with the dose of Dietzia and not ELISA values; the higher the dose, the lower the shedding and vice versa---supporting conclusion reached previously with different cows [11,13,14].

**Figure 3:**
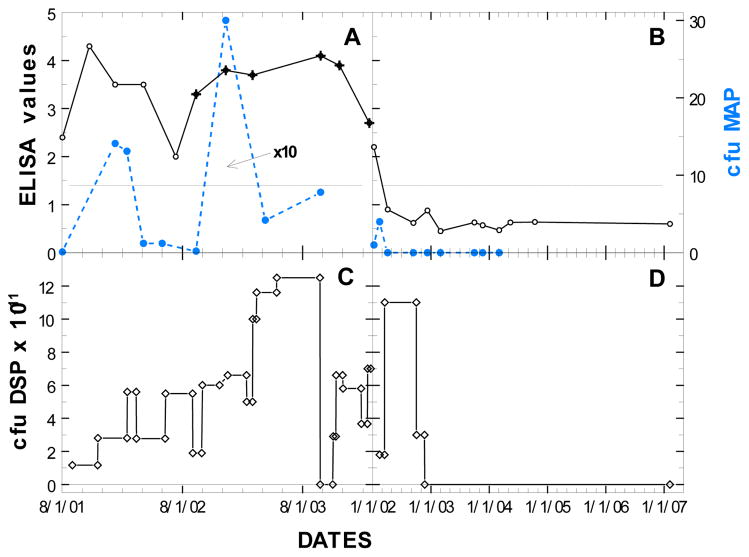
Paratuberculosis parameters of two cows with early, initial stages of disease at different doses of Dietzia. Cow # 21 (panels A, C) eventually succumbed with disease, whereas cow #229 (panels B, D) was cured.

In Figure 3, ELISA, AGID, and fecal shedding are shown at different doses of Dietzia for two cows {#21--panels A-C--weighing 1300 #s and #229–panels B-D--weighing 900#s (ELISA values only for #229 were previously shown in ref 14)} that were started on treatment at an earlier stage of disease than that of cow #R100 (low ELISA values and low or no fecal shedding). As shown for #21, at high doses of *Dietzia*, there were low levels of MAP shed, whereas when doses were low, shedding was high. Changes in ELISA values on the other hand were associated more with extended changes in MAP shedding, rather than with the dose of Dietzia. When shedding was low or absent, ELISA values trended lower; if shedding was significant, high ELISA values and positive AGID were found. In contrast to cow #21, cow #229 was maintained on a very high dose of Dietzia based on her size for almost 7 months. Longitudinally, her initial ELISA value declined very rapidly. Once both the OD values became (and remained) less than 1.4 (considered negative) and fecal MAP was/remained undetectable for months, she was considered cured and treatment was discontinued. She was eventually culled from the herd four years after termination of treatment with no evidence of paratuberculosis.

**Figure 4:**
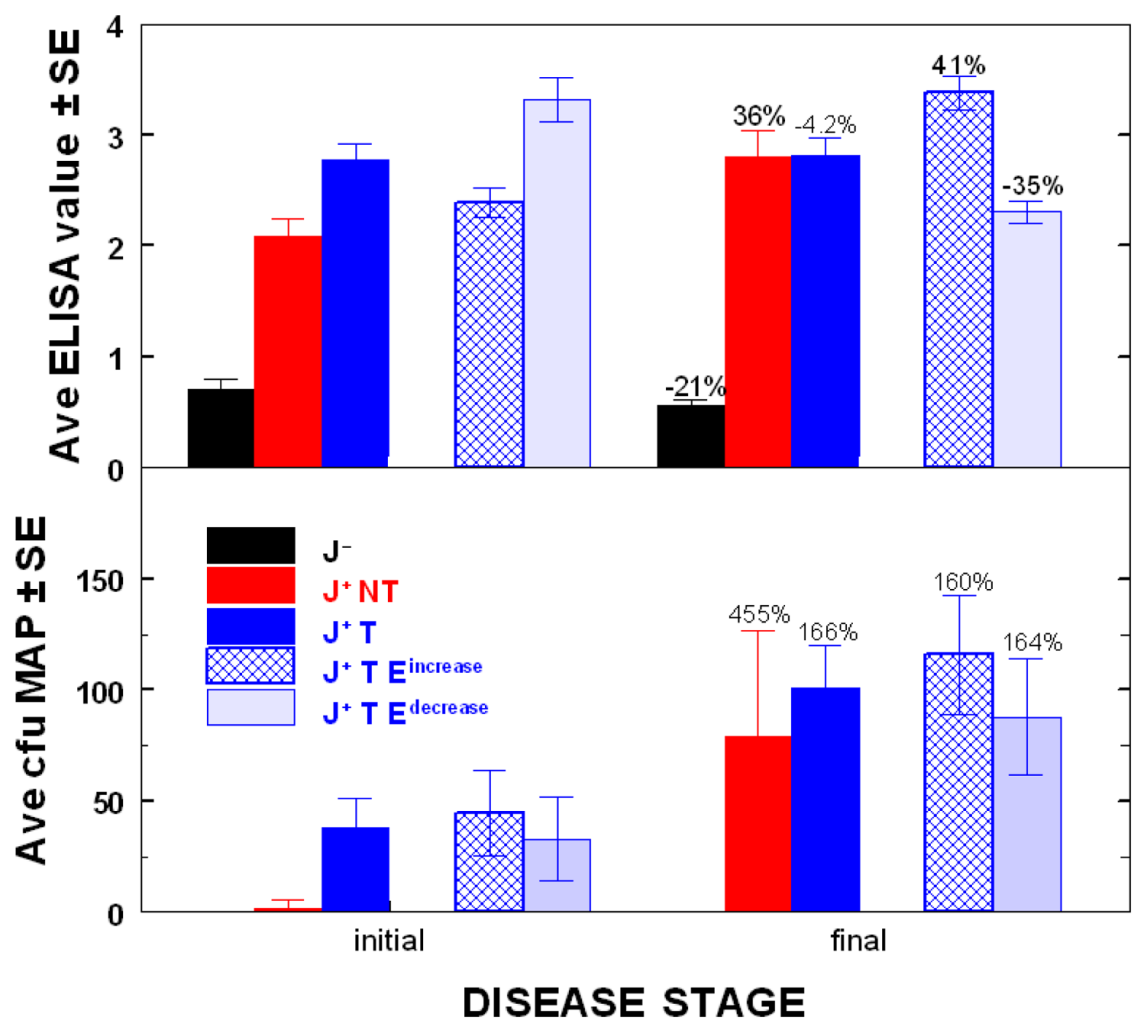
Changes in mean ELISA OD values and fecal MAP of paratuberculosis-free and –positive cows, treated and not treated. Initial and final ELISA OD values are in panel A and fecal values in Panel B. J-- = Johne’s disease-free (n=10); J^+^ NT = Johne’s disease positive, not treated (n=9) J^+^ T = Johne’s disease positive, treated (n=49); J+ T E^increase^ = Johne’s disease. positive with longitudinal increasing ELISA values (n=20); J^+^ T E^decrease^ = Johne’s disease positive with longitudinal decreasing ELISA values (n=22) Percentages shown for each group indicate changes relative to initial values.

Because longitudinal ELISA values for paratuberculosis, treated cows, remained either unchanged, decreased, or increased, clinical outcomes of animals in the latter two categories were compared (the seven with ELISA values that did not change because all were at a terminal stage of disease, were not included). The mean initial and final ELISA OD values and fecal MAP values for cows that were paratuberculosis-free (n = 10), paratuberculosis-positive, not-treated (n = 9), and positive, treated animals (n = 49) that had either longitudinally increasing (n = 20) or decreasing (n = 22) ELISA values are shown in Figure 4. Even though the mean initial ELISA OD value of paratuberculosis, treated cows was slightly higher (p = 0.0094) than that of those not treated, final values for both were indistinguishable (difference of 1.8%). In other words, over a lifetime, the mean ELISA OD value of nontreated cows increased 36% whereas that of those treated, remained essentially unchanged (−4.2%). As also shown, the mean initial ELISA OD for the decreasing longitudinal group was actual higher (indicating more advanced disease) than that of the increasing group and yet the final mean ELISA OD was 35% lower than that at initiation, whereas it was 41% higher for those with increasing values; both changes highly significant (P = <0.001).

Shown in Figure 4B, initial fecal shedding was much lower in the non-treated group compared to the treated group, whereas final shedding of the two groups was not significantly different (P = 0.27). Thus, MAP shed by the non-treated group increased 455%, whereas all treated groups increased equally (166%, for all animals, and 160% and 164% for those with increasing or decreasing ELISA trends---none predictive of progression/regression of disease). The large discrepancy in initial MAP shed was a consequence of the decision to assign animals to either the treated group or the non-treated group based on initial ELISA values instead of values for MAP shed. This decision was made because these two test-parameters were at most, only loosely associated [12] and because it requires 60–90 days of culture to obtain an accurate fecal value---time during which, if treatment was the choice, it needed to be ongoing.

**Figure 5:**
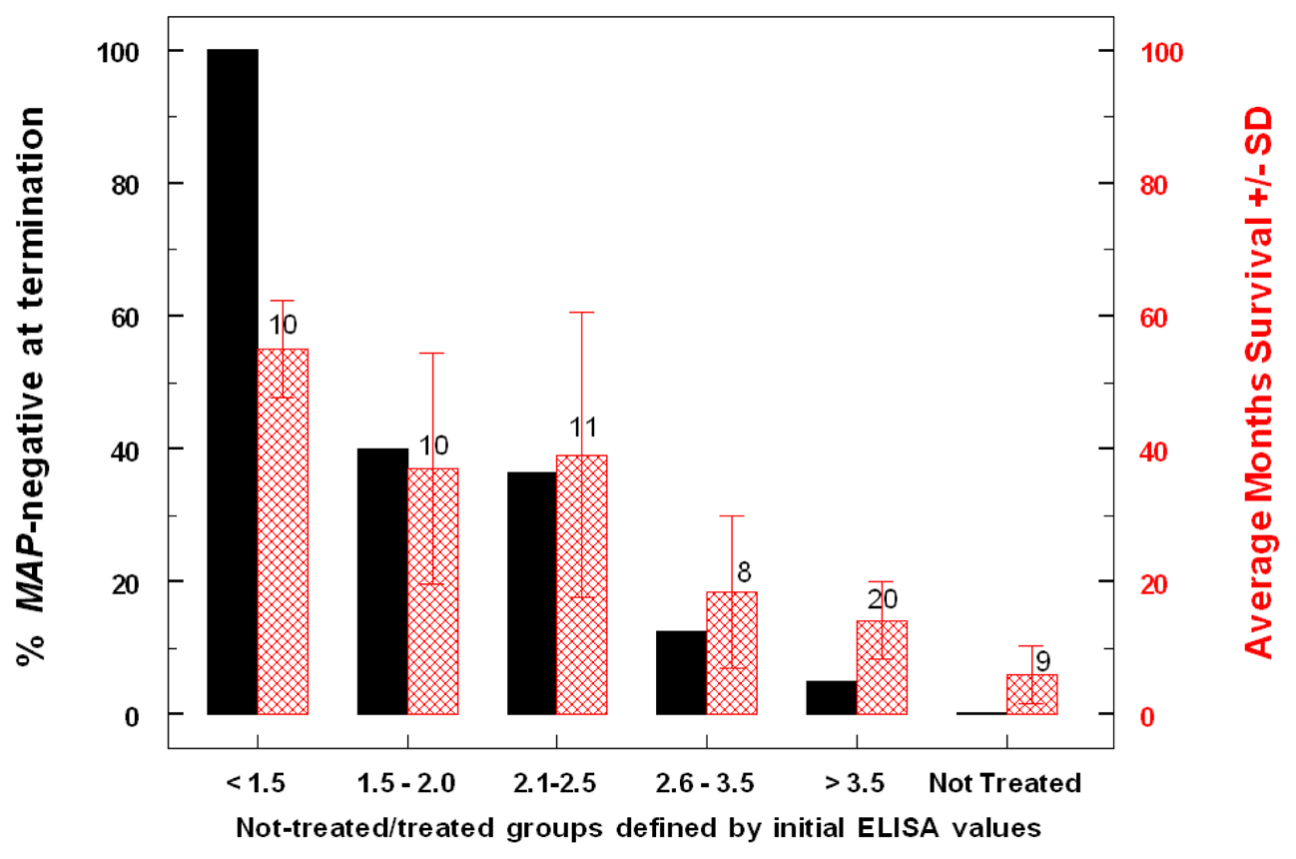
Association of percent cures and longevity with ELISA OD values of treated and non-treated, paratuberculosis-free and paratuberculosis-positive adults. Values over each group indicate the number of animals.

Figure 5 shows the curative levels achieved by treatment of animals at different stages of disease (arbitrarily grouped by incremental initial ELISA OD values). In addition, mean longevity is also shown for each of the groups. As the ELISA OD values at which treatment was initiated increased (presumed to indicate severity of disease), the percentage of animals cured and the length of survival declined; at ELISA OD values > 2.5 only 2 of 28 were cured. The percent cured (>38%) in the treated Johne’s-disease groups with ELISA ODs of 1.5–2.1 and 2.1–2.5 was significantly different from that of the paratuberculosis-positive, not treated group and the other treated groups. Survival of the paratuberculosis-free group was not significantly different from that of either the 1.5–2.0 group (p=0.41) or the 2.1–2.5 group (p=0.17), and these two groups did not differ from one another (p=0.47). Similarly, the survival of the not treated, MAP-positive group was not significantly different from that of the treated ELISA OD >3.5 group (p=0.087), whereas length of survival of the 2.6–3.5 group was significantly longer than that of the >3.5 group (p=0.019), but not different from that of the 2.1–2.5 group (p=0.15).

## Discussion

Johne’s disease in ruminants, such as cattle, sheep, goats, and even wild-life, has many manifestations in common with CD [16–19], including debilitating diarrhea in cattle. As with CD there are no preventive/curative therapies for animals with Johne’s disease, outside Dietzia therapy [11,13,14]. The Johne’s disease, etiologic agent is *Mycobacterium avium* subspecies *paratuberculosis* (MAP). Natural exposure of cattle to MAP, which usually results in clinical disease at >2 of age, occurs primarily in utero and/or by oral ingestion of MAP by neonates [23 and ref therein]. For disease to be manifested, infection with MAP and intestinal inflammation are required. Interestingly, MAP is present in a number of different human diseases; i.e., Crohn’s disease [24–28], ulcerative colitis [28], irritable bowel syndrome [28], sarcoidosis [29], type-1 diabetes mellitus [30–32], Blau syndrome [33], and multiple sclerosis [34]. However, the consequences of MAP infection—cause, perpetuate, bystander--is highly controversial and remains to be unequivocally clarified. Perhaps a question almost as intriguing is: What is the significance of MAP in control, seemingly healthy individuals? Recently, an astonishingly 33% of such individuals were found [35] to be infected with MAP! The presence of MAP at a single time point in such a large percentage raises a question as to what extent is the general population exposed to (infected with) MAP at some point in their life? It was recently suggested [14,15] that this number is grossly under-estimated, and that exposure/infection could be at an epidemic level. Based on this, in conjunction with the presence of MAP in patients with numerous, seemingly unrelated diseases, many have called for a more concerted effort towards eliminating MAP from the food chain, irrespective of its yet, to be defined role.

It has been proposed that therapy directed against both a bacterial etiology agent and against inflammation may be a more fruitful approach for controlling Crohn’s disease than conventional monotherapies [36–38]. Strategies aimed at restoring the intestinal microbial balance—fecal bacteriotherapy [39], probiotics [40], and numerous antibiotic regimens [41,42]—have been employed with some success. These modalities seem too have fewer side-effects than conventional treatments that broadly suppress inflammation and/or innate immunity [43]. Two protocols that have resulted in ‘cures’ of CD---a side benefit of immune ablation, allogeneic/autologous marrow reconstitution used for treating unrelated diseases [44], and a single case of prolonged anti-Mycobacterial triple antibiotic therapy [45] have been reported. Only the latter was tested for and resulted in elimination of detectable MAP and is the only case demonstrating an association of CD resolution with loss of detectable MAP. In two other studies in which triple therapy was used, opposing results were found—a retrospective review of 39 severely ill IBD patients found long lasting benefits [46] suggesting CD is driven by infection, whereas in the other, no benefits were found [47,48]. That no, or only palliative, benefits were found should have been anticipated based on the palliative benefits achieved with a similar anti-mycobacterial antibiotic therapy of paratuberculosis goats and cattle—once treatment was stopped, clinical disease again progressed because MAP was not eliminated [49–51]. Thus, one needs to question whether the opposing differences reported for humans have any relevance for the question of whether MAP does or does not play a significant role in the pathogenesis of Crohn’s disease, since in neither study was the presence/absence of MAP assessed before, during, or after termination of treatment! Perhaps a similar study in which anti-mycobacterial therapy is replaced with Dietzia (which unlike anti-mycobacterial therapy eliminated MAP in cattle) will lead to a different outcome.

While a number of human gastrointestinal, hepatic and other diseases [40] have been successfully treated with organisms functioning as probiotics, trials with a variety of common probiotics have not proven effective for CD [52,53]; in fact, Lactobacilli have proven completely ineffective [54,55]. Thus, the question: What distinguishes Dietzia, used as a probiotic, from those considered more traditional— Lactobacillus, Bifidobacterium, and Streptococcus? Dietzia is an aerobic, Gram-positive, nonsporing, actinobacteria, and a close relative of MAP [56]. The mechanism by which Dietzia curtailed/reduced fecal MAP shedding in cattle remains unknown. The consequence of such a reduction, however---subsequent decline of serum antibodies resulted in ‘cured’ animals—was that anticipated. A property unique to Dietzia is it, unlike other probiotics, inhibited the growth in culture of the pathogen that causes disease [21]; namely the etiologic agent of Johne’s disease. Based on these results, potential mechanisms are: (a) close intracellular encounters of MAP and Dietzia (in macrophages/dendritic cells?) may be enhanced by cross-reactive antibodies specific for epitopes they share (William D. Richards, personal communication and REC, unpublished), thus facilitating opsonization of Dietzia/MAP by phagocytes already possessing the other organism, MAP/Dietzia; (b) Dietzia, when in close intracellular proximity to MAP, may either inhibit its growth via competition for a nutrient(s), destroy it directly or indirectly, and/or prevent/reverse MAP elaborated factors that interfere with normal, host cell signaling defense machinery [57]; (c) N-acetyl-MDP glycan moiety of Dietzia’s cell wall, compared to the N-glycolyl MDP of MAP [58,59], being less potent and efficient at activation of NOD2, may permit Dietzia-induced protective responses that counter MAP’s inhibitory responses [60] without exacerbating inflammation {NOD2 is a gene implicated in Crohn’s and Johne’s diseases [61,62] that, when activated, leads to production of proinflammatory cytokines [63,64]}; (d) steroid disruption of normal phagocytic cellular activity/integrity could impair activation of immune responses and/or could impair potential MAP killing (in fact, the level of MAP shed in bovine feces [14] and that present in human blood and intestinal tissues increased after steroid treatments [35,65]; and (e) in the absence of co-infection and/or if macrophage/phagocytic functions are impaired, MAP levels would continue to increase (as was found) and enhance disease. The most intriguing aspect of these alternatives is, that for Dietzia to effectively reduce and eliminate MAP in cattle, some aspect of immune reactivity (either/or functional phagocytes, B-cells, T-cells) plays a crucial role in reducing MAP levels, which in turn resulted in a decline of MAP-specific antibody and regression of disease [14]; immune activity in the absence of Dietzia did/does not lead to cures. Thus, the perplexing problem: How can inflammation be curtailed sufficiently to allow healing and restitution of normal intestinal function while allowing Dietzia therapy to reduce/eliminate MAP? This scenario is most relevant for advanced stages of disease since the level of MAP in animals at early stages of disease is low, and in many cases, undetectable.

Potential benefits of Dietzia for patients with IBD (initial emphasis on CD) are permanent amelioration of diarrhea and/or attainment of long-term remissions/cures. To be effective long-term, two processes must be curtailed: (a) inflammation of the intestine, presumed to be due to an immune response to an etiologic agent (MAP in cattle), and (b) eradication of the etiologic agent stimulating the immune system. Since the crucial effect of Dietzia in cattle was to curtail/reduce MAP, reduction of MAP in CD patients infected seems feasible and is an obvious target-test population. Thereafter, assessment of any association of MAP changes to clinical symptoms/outcomes would be instructive. Thus, the question: Which ante-mortem assay will be most informative as a means to monitor MAP changes and at what stage of CD could a Dietzia-benefit most likely be achieved?

Previous findings [13] indicated that for cattle, the most informative assay to monitor clinical progression/regression was longitudinal changes in ELISA OD values; changes in fecal MAP levels, being extremely variable, were not informative (as also shown herein). Cures were only found in animals that had longitudinally decreasing ELISA values; treated animals with increasing or unchanging values were never cured, even though survival was extended relative to those not treated. Moreover, sustained, longitudinally declining values occurred only after either, continued reduction of fecal MAP, or if MAP was not detected at the time treatment commenced, its absence was maintained; i.e., antibody levels depended upon synthesis driven by MAP antigens plus their normal biological half-life. Thus, if it is presumed that MAP plays a role in CD, infected patients projected to benefit the most from a Dietzia treatment are those that are at an early stage of disease with low levels of systemic MAP. Since many Crohn’s patients are MAP seropositive [35,65–67], a human, MAP specific ELISA assay [66,67] should permit reliable monitoring of changes in MAP, allow correlating ELISA results to cultureable, systemic MAP and to clinical progression of disease---the latter defined by curtailment of diarrhea as an early benefit and by standard clinical parameters such as colonoscopy/biopsies for long term benefit.

## Conclusions

In summary, based on previous findings [14] and those herein, that: (a) MAP in feces (and presumably systemically) need to be reduced to extremely low or undetectable levels prior to achieving any decline in serum antibodies as detected by Allied Monitor, Inc. in a MAP specific ELISA; and (b) ELISA OD values need to longitudinally decline to levels considered negative prior to an animal going on to live a normal, uneventful, Johne’s disease-free life; i.e., the cow was presumably cured. These Dietzia therapeutic benefits for Johne’s diseased animals and the similarity of Johne’s and Crohn’s diseases, are sufficiently compelling to warrant undertaking a clinical Dietzia trial with Crohn’s patients, and to relate effectiveness to both the presence/absence of MAP and to genes associated with Crohn’s disease, such as *NOD2*. Because viable Dietzia has no adverse side effects and was nonpathogenic when orally administered to adult cows (daily for up to 2 years) or calves starting at birth for 60 days [14,15], as well as when injected IP into normal or immunodeficient mice [14], it is anticipated that it will also be safe for oral consumption by humans---a tremendous advantage relative to other present day therapies.
